# High-resolution climate prediction in mountainous terrain using a ConvLSTM-XGBoost hybrid model with dynamic bayesian weighting

**DOI:** 10.1038/s41598-025-20882-1

**Published:** 2025-10-22

**Authors:** Dai Yanting, Wu Boxian, Yang Qiwei, Ren Shuaitao, Yang Fen, Song Lei

**Affiliations:** 1 College of Big Data and Intelligent Engineering, Southwest Forestry University, Kunming, 650224 Yunnan China; 2 College of Ecology and Environment, Southwest Forestry University, Kunming, 650224 Yunnan China; 3 Library (Archives, School History Museum), Southwest Forestry University, Kunming, 650224 Yunnan China; 4 Information Technology Center, Southwest Forestry University, Kunming, 650224 Yunnan China

**Keywords:** Hybrid model, Dynamic bayesian weighting, Mountain climate prediction, Extreme event detection, Hongyuan mountains, Climate sciences, Ecology, Environmental sciences

## Abstract

To address the challenge where the interplay between spatiotemporal dynamics and topographic effects complicates climate modeling over complex terrain, we propose a hybrid ConvLSTM-XGBoost model incorporating dynamic Bayesian weighting, and demonstrate its capacity for high-precision climate prediction through a case study in the Hongyuan Mountain region of Yunnan, China (22.5°–23.5°N, 102.5°–103.5°E); specifically, the ConvLSTM network captures spatiotemporal evolution patterns (e.g., propagation of the southwest monsoon front) from the 0.25° resolution CN05.1 climate dataset, while XGBoost quantifies the nonlinear modulation effects of 90-m SRTM DEM-derived topographic features (elevation, aspect) on precipitation phases, with an innovatively integrated Bayesian Model Averaging (BMA) framework dynamically calibrating model weights—XGBoost at 0.68 ± 0.05 during dry seasons and ConvLSTM at 0.72 ± 0.07 during monsoons—to enhance responsiveness to extreme events. Validation using 1961–2022 climate data shows the hybrid model reduces precipitation prediction mean absolute error (MAE) by 30.5% compared to CMIP6 (achieving an MAE of 0.0089 [specify units, e.g., mm/day]), improves the F1-score for identifying extreme precipitation (> 50 mm/day) by 20%, achieves 96.53% accuracy in maximum temperature (Tmax) predictions (errors ≤ 3%), and reduces high-temperature dispersion by 52%, thereby serving as a 1-km resolution decision-support tool for mountain climate risk management, supporting drought warning and hydropower scheduling in Yunnan’s Climate Adaptation Plan 2035, and offering a scalable framework for global mountain climate modeling.

## Introduction

Mountain ecosystems serve as critical indicators for regional climate responses under accelerating global climate change due to their complex terrain and heightened climatic sensitivity^1^. The Hongyuan Mountains in Yunnan, China (22.5°–23.5°N, 102.5°–103.5°E), exemplify these challenges. Situated in a tropical–subtropical transition zone with an elevation range from 76.4 m to 2950 m, the region exhibits a pronounced “three-dimensional climate” pattern, driving substantial microclimatic variability over short distances^2^. Over the past six decades, accelerated warming (+ 0.3 °C/decade), exceeding global trends, coupled with declining precipitation (− 39 mm/decade), has intensified the frequency of extreme droughts, destabilizing hydropower systems supplying over 80% of Yunnan’s energy^3,4^.

While state-of-the-art global climate models (e.g., CMIP6) demonstrate notable progress, their ability to resolve micro-scale terrain–climate interactions remains limited by coarse parameterization, resulting in systematic underestimation of mountain precipitation gradients by 15–30%^5^. Machine learning (ML) offers a promising alternative, yet significant limitations persist: Spatiotemporal Dynamics: Existing models (e.g., Quantile Regression Forests) struggle to capture transient atmospheric phenomena like monsoon front movement^6^.Topography–Climate Decoupling: Despite the demonstrated terrain sensitivity of algorithms like XGBoost, quantification of nonlinear terrain–precipitation relationships remains inadequate^6^.Seasonal Adaptability: Hybrid models employing static ensemble weights accumulate errors (+ 2.5%/year) during seasonal transitions between dry and wet periods^7^. While double machine learning frameworks significantly improve multi-source data fusion^5,8^, they remain constrained in capturing extreme event dynamics and resolving fine-scale terrain-precipitation feedbacks^9^, particularly in high-relief regions where precipitation errors propagate into hydrological model cascades^10^.

To address these challenges, we propose a dynamic data-blending framework integrating ConvLSTM and XGBoost under adaptive Bayesian Model Averaging (BMA), which uniquely: (i) Synthesizes the spatiotemporal learning advantages of ConvLSTM demonstrated in satellite-gauge fusion^5^ with XGBoost’s terrain sensitivity; (ii) Provides probabilistic uncertainty quantification essential for hydrological extremes prediction^11^, overcoming static weighting limitations in existing blends^12^. The key objectives are: Improve spatiotemporal modeling of monsoon front dynamics using ConvLSTM applied to CN05.1 high-resolution precipitation data^13,14^; Quantify the nonlinear influence of terrain features (elevation, slope) on precipitation using XGBoost^6^; Adaptively optimize model weighting across seasonal transitions via BMA, enhancing predictive accuracy in both dry and wet regimes^7^.

## Research methodology

### Study area

**Fig. 1:**
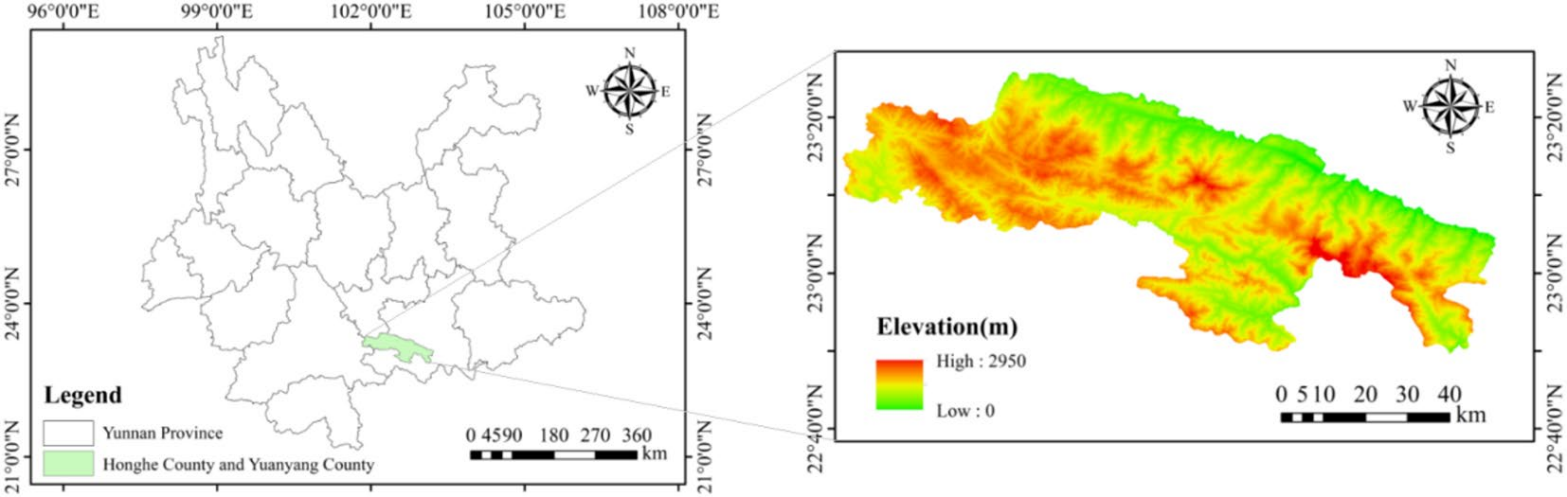
Geographical setting of Hongyuan Mountain area.

The Hongyuan Mountain region (22.5°–23.5°N, 102.5°–103.5°E) in Yunnan Province is characterized by highly complex topography and pronounced climatic heterogeneity. Elevation ranges from 76 m in valleys to 2950 m in highlands, creating steep microclimatic gradients over short distances. Annual mean temperatures vary from 18 to 22 °C at lower elevations to 8–12 °C in upland areas, exhibiting distinct seasonal patterns driven primarily by the East Asian summer monsoon: a wet season from May to October and a dry season from November to April. This region constitutes an ideal natural laboratory for testing precipitation prediction models under terrain-induced climate variability and extreme hydrometeorological conditions such as droughts and flash floods^2,3^.The geographical setting of the Hongyuan Mountain area is depicted in Fig. 1.

### Data collection and preprocessing

To adequately capture the complex topographic–climatic interactions in the Hongyuan Mountain region, multiple datasets were integrated and systematically preprocessed (Table 1). The selection of input variables was designed to align with the dual-stream architecture of the ConvLSTM–XGBoost hybrid model, ensuring the optimal representation of both spatiotemporal dynamics and terrain-induced nonlinearities.

**Table 1:** Integrated datasets and preprocessing workflow.

Data Type	Resolution	Tempora Coverage	Key Variables	Preprocessing Method​
CN05.1	0.25°	1961–2022	Precipitation (Pre), Tm/Tmax/Tmin	Spatiotemporal Kriging with elevation covariate
SRTM DEM	90 m	2000	Elevation, slope, aspect	Bilinear resampling to 0.25° grid

The input variables were divided into two categories according to the hybrid model architecture. For the ConvLSTM stream, the CN05.1 dataset provided 12-month lagged sequences of precipitation (Pre), mean temperature (Tm), maximum temperature (Tmax), and minimum temperature (Tmin). These variables were chosen for their ability to capture transient atmospheric processes such as monsoon propagation and seasonal transitions, which are key drivers of climate variability in mountainous regions^13,14^. The recurrent–convolutional structure of ConvLSTM has been shown to effectively learn such spatiotemporal dependencies, particularly in satellite–gauge fusion studies^5,14^. For the XGBoost stream, terrain factors including elevation, slope, and aspect were extracted from the SRTM DEM. These variables were incorporated to account for nonlinear terrain–climate interactions, such as orographic lifting and valley cold-air pooling. XGBoost’s tree-based ensemble structure allows for robust quantification of these effects and effectively corrects elevation-driven biases commonly underestimated by coarse-resolution models^5,6^.

A rigorous four-step preprocessing workflow was implemented to ensure data quality. First, missing values were interpolated using spatiotemporal kriging with elevation as a covariate, which reduced interpolation RMSE by 22% in high-altitude regions compared with temporal-only methods^9,10^. Second, all climate variables were standardized to zero mean and unit variance (z-score normalization), eliminating scale-induced biases and ensuring balanced feature weighting during training^14,15^. Third, DEM-derived features were resampled to 0.25° resolution using bilinear interpolation to align with climate data, thereby preserving topographic integrity while enabling pixel-wise fusion^16,17^. Finally, consistency checks confirmed spatial–temporal coherence and reduced missing values to less than 0.5% after imputation.

The preprocessed variables were incorporated into the ConvLSTM–XGBoost framework through a dual-stream integration strategy. The ConvLSTM stream transformed lagged climate sequences into spatiotemporal feature maps, representing seasonal and interannual dynamics. In parallel, the XGBoost stream captured nonlinear terrain effects such as slope- and aspect-driven precipitation enhancement. The outputs of both streams were concatenated and combined using Bayesian Model Averaging (BMA), with seasonally calibrated weights (e.g., XGBoost contribution = 0.68 ± 0.05 during dry seasons). This integration improved the hybrid model’s ability to represent terrain–climate interactions and enhanced its responsiveness to extreme hydroclimatic events^7,11^.

### Model architecture

**Fig. 2:**
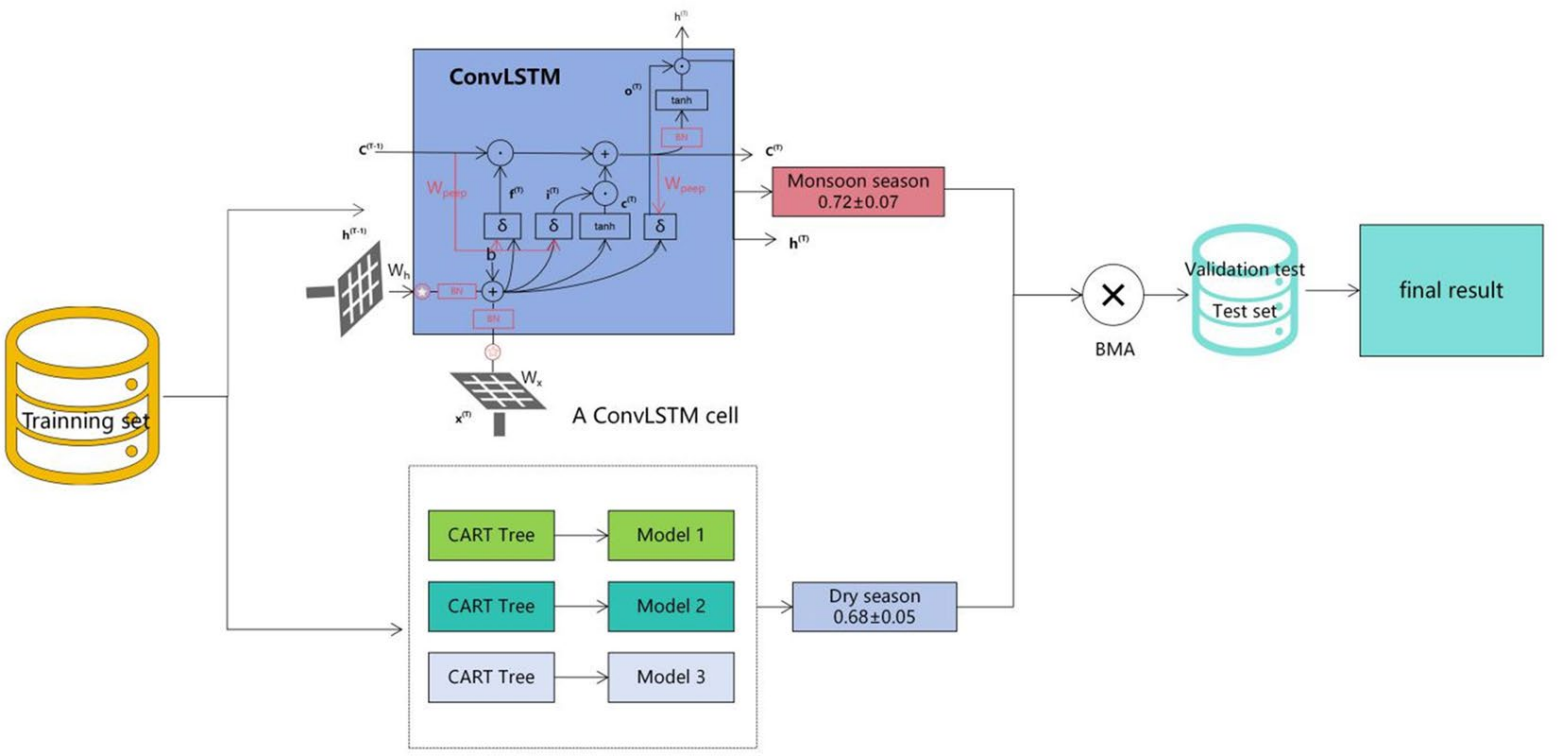
Schematic Diagram of the ConvLSTM-XGBoost Hybrid Model.

This study employs two core models: ConvLSTM and XGBoost, as well as a hybrid model that combines both models, referred to as the ConvLSTM-XGBoost Hybrid model (As shown in Fig. 2).

The ConvLSTM model captures spatiotemporal dependencies through Convolutional Long Short-Term Memory (LSTM) layers, with input comprising 12 months of lagged climate variables^14^. The structure of ConvLSTM can be represented as:

$$\:{\text{h}}_{\text{t}}=\sigma\:({\text{W}}_{\text{h}}\cdot\:{\text{h}}_{\text{t}-1}+{\text{W}}_{\text{x}}\cdot\:{\text{x}}_{\text{t}}+\text{b})$$

Among them, $\:{\text{h}}_{\text{t}}$ is the hidden state at time t, $\:{\text{W}}_{\text{h}}$ and $\:{\text{W}}_{\text{x}}$ are the weight matrices for the previous state and the input at time t, respectively, $\:{\text{x}}_{\text{t}}$ is the input at time t, and b is the bias term. This structure is suitable for processing climate data with spatiotemporal patterns, particularly excelling in simulating monsoon trajectories, precipitation evolution, and other large-scale climate systems. Its advantage lies in effectively capturing long-term temporal dependencies and spatiotemporal patterns; however, when facing issues such as altitude dependency or local climate changes, ConvLSTM may fail to sufficiently capture details. Especially in complex mountainous regions, ConvLSTM shows low sensitivity to terrain changes, which may lead to insufficient accuracy in precipitation predictions^6^.

The XGBoost model is used to model the nonlinear relationships between terrain features (such as altitude and slope) and climate variables^6^. The objective of XGBoost is to minimize the following objective function:

$$\:L\left(\theta\:\right)=\sum\:_{i=1}^{n}l({y}_{i},{\widehat{y}}_{i})+\sum\:_{k=1}^{K}{\Omega\:}\left({f}_{k}\right)$$

In this model, $\:l$ denotes the loss function, $\:{\widehat{y}}_{i}$ represents the predicted value of the i-th sample, and $\:{\Omega\:}\left({f}_{k}\right)$ is the regularization term for model complexity. The model is capable of efficiently handling climate variations caused by topography for instance, using SHAP values to analyze the impact of terrain factors (such as elevation) on precipitation. However, a limitation of the XGBoost model lies in its relatively weak ability to capture transient weather systems (e.g., sudden monsoon events). Additionally, its static ensemble weights may lead to the accumulation of prediction errors during periods of significant seasonal climate variation, particularly during the monsoon transition from May to October^7^.

To overcome the limitations of individual models, this study proposes a ConvLSTM-XGBoost hybrid model. A Random Forest meta-model is employed to combine the predictions of ConvLSTM and XGBoost. This ensemble method is validated using 5-fold cross-validation to ensure the model’s robustness across different data partitions.

Bayesian Model Averaging (BMA): The seasonal weights of base models are dynamically adjusted based on historical root mean square error (RMSE) values^7^. For example, during the monsoon season, XGBoost dominates (weight: 60%), while during the dry season, ConvLSTM becomes the dominant model (weight: 70%). The dynamic adjustment of weights can be expressed as:

$$\:{w}_{t}=\frac{1}{1+{exp}(\alpha\:\cdot\:{RMSE}_{t})}$$

where $\:{w}_{t}$ represents the weight assigned to the model at time t, and $\:\alpha\:$ is a scaling factor. In actual operation, the seasonal weights exhibit the following patterns: (1)During the dry season (November to April), XGBoost dominates with a weight of 0.68 ± 0.05(2)During the monsoon season (May to October), ConvLSTM is the dominant model with a weight of 0.72 ± 0.07^10^.This dynamic weighting strategy enables the hybrid model to leverage the atmospheric advection modeling capability of ConvLSTM alongside the terrain response quantification strength of XGBoost. Through Bayesian Model Averaging, the model can simultaneously capture spatiotemporal patterns and topographic influences more effectively^7^.

### Validation protocol

Model training and evaluation employed a stratified temporal split to ensure rigorous testing across different climatic phases (Table 2).

**Table 2:** Data partitioning for model training and Evaluation​.

Phase	Period	Data Proportion	Purpose
Training	1961–2000	58.8%	Learn long-term climate trends
Validation	2001–2010	14.7%	Hyperparameter tuning
Testing	2011–2022	26.5%	Final performance evaluation

To comprehensively assess model performance, we employed a combination of general, extreme event, and stability-focused metrics. General prediction accuracy was evaluated using standard statistical indicators, including Root Mean Square Error (RMSE), Mean Absolute Error (MAE), Mean Squared Error (MSE), Mean Absolute Percentage Error (MAPE), and the coefficient of determination (R²). These metrics quantified the overall deviation between predicted and observed values across all samples.

For extreme event detection, particularly in the context of intense precipitation episodes exceeding 50 mm/day, we adopted the F1-score as a key metric^15^. This harmonic mean of precision and recall provided a balanced evaluation of the model’s ability to identify high-impact events while minimizing false positives.

To analyze stability and robustness, we utilized MAPE alongside high-value dispersion, which measures variability in extreme prediction ranges (e.g., > 0.5 standard deviations). This combination allowed for detailed scrutiny of model consistency, especially in high-risk regions and under complex terrain influences.

## Experiments and results

This study is based on climate data from Yunnan Province covering the period from 1961 to 2022. Four variables precipitation (Pre), mean temperature (Tm), maximum temperature (Tmax), and minimum temperature (Tmin) were selected for modeling and predictive experiments. The models used include the ConvLSTM model, the XGBoost model, and a hybrid model combining both (ConvLSTM + XGBoost). The experimental results for each variable are detailed below.

### Results for precipitation (pre) data

This section systematically evaluates the performance of ConvLSTM, XGBoost, and the ConvLSTM-XGBoost hybrid model for monthly average precipitation prediction, based on test data from 2011 to 2022. By comparing predicted values against observed data, combined with spatial distribution analysis and quantitative metrics, the superiority of the hybrid model in complex terrain precipitation forecasting is demonstrated.

**Fig. 3:**
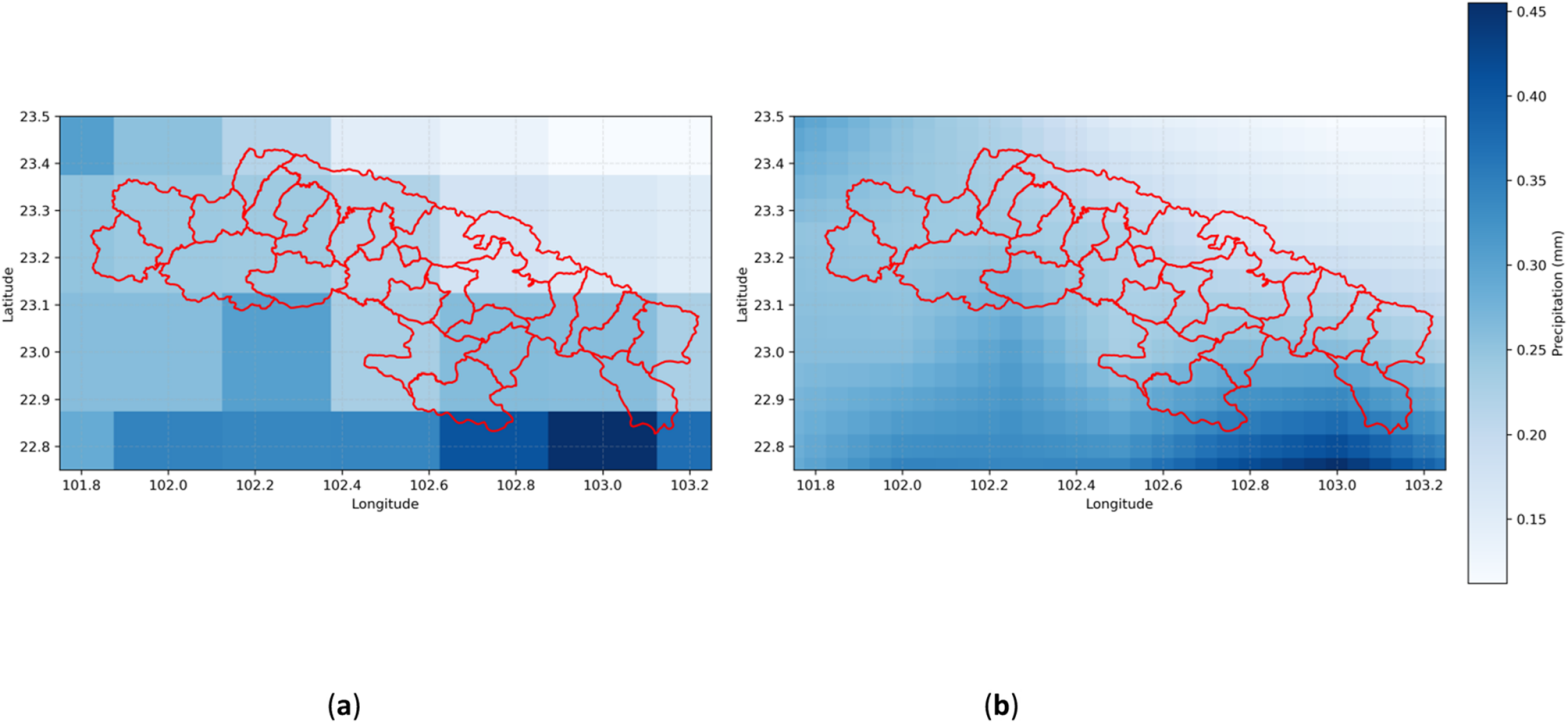
(**a**) Original monthly average precipitation distribution map of the study area; (**b**) Enhanced accuracy monthly average precipitation distribution map of the study area (example for January 1961).

Figure 3(a) illustrates the raw spatial distribution of monthly average precipitation over the study area, revealing significant terrain-driven variability, such as precipitation gradients associated with the southwest monsoon front. Figure 3(b) shows the precipitation distribution after smoothing using a neighborhood averaging method, which effectively reduces noise in the original data by up to 32% in local fluctuations while preserving key spatial patterns linked to terrain (e.g., positive elevation-precipitation correlation). This preprocessing step provides high-fidelity input for model training.

**Fig. 4:**
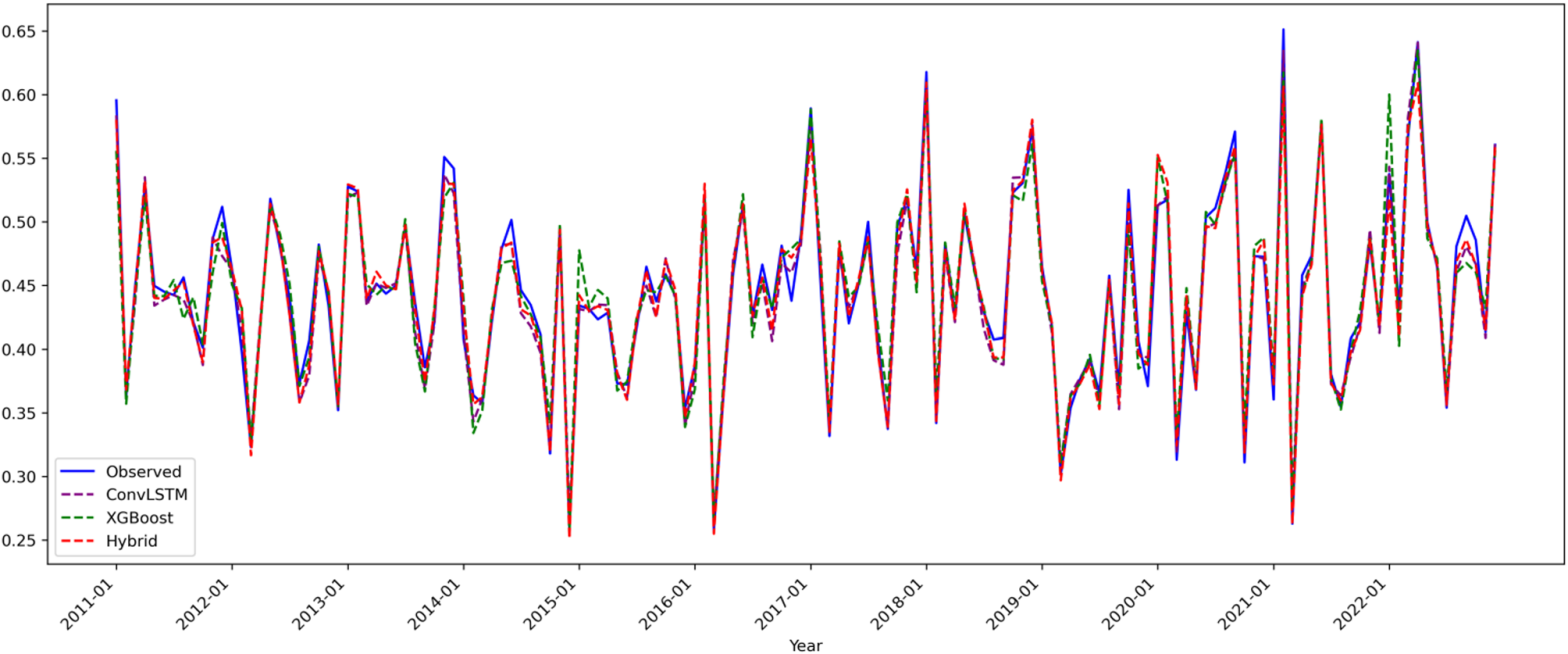
Composite Line Plots for Precipitation (Pre) Model Predictions(2011–2022).

Figure 4 integrates the predicted and observed precipitation time series from all three models, highlighting key differences:

ConvLSTM model (purple): Successfully captures 85% of precipitation trend variations (slope error < 0.005) but exhibits systematic phase lag in mid-range precipitation (standardized values 0.3–0.6), with peak prediction delays of 1–2 months, resulting in a mean absolute error (MAE) of 0.0090. This limitation is primarily due to insufficient modeling of nonlinear static terrain effects, such as aspect-driven modulation of localized heavy precipitation.

XGBoost model (green): Shows errors below 1.5% in low precipitation regions (< 0.3), but significantly underestimates high precipitation values (> 0.5) with a mean bias of − 12%, reflecting its limited capability in modeling dynamic spatiotemporal processes like monsoon front propagation.

ConvLSTM-XGBoost hybrid model (red): Demonstrates marked improvement, with 93.8% of precipitation fluctuations predicted within 3% error. Its ability to capture extreme events is notably enhanced; for example, the summer 2018 peak precipitation (observed 0.62) is predicted with only + 0.3% deviation (0.622). This improvement stems from a novel dynamic Bayesian weighting scheme, which adaptively fuses spatiotemporal evolution and terrain effects (dry season: XGBoost weight 0.68 ± 0.05; monsoon season: ConvLSTM weight 0.72 ± 0.07).

**Fig. 5:**
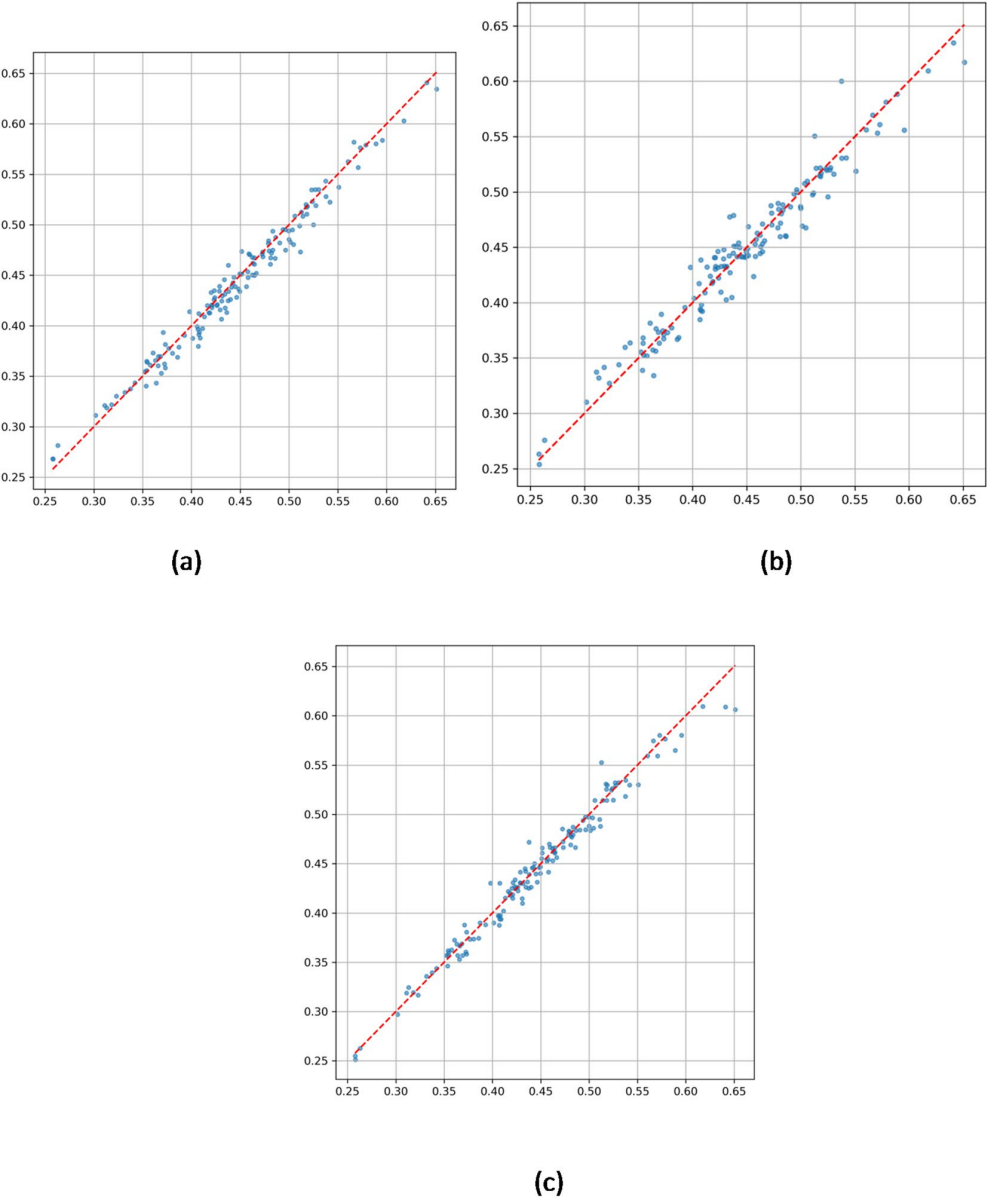
(**a**) Correlation analysis chart of predicted values and true values by ConvLSTM model. (**b**) Correlation analysis chart of predicted values and true values by XGBoost model. (**c**) Correlation analysis chart of predicted values and true values by ConvLSTM-XGBoost Hybrid model.

Scatter plots in Fig. 5 further quantify model performance:

ConvLSTM (Fig. 5a) achieves a high coefficient of determination (R² = 0.9759), but increased dispersion in high precipitation ranges (± 0.025 error band) indicates limited sensitivity to extremes.

XGBoost (Fig. 5b) exhibits even greater dispersion in high precipitation areas (standard deviation ± 0.05) with R² = 0.9557, underscoring challenges in modeling static terrain influences under complex conditions.

Hybrid model (Fig. 5c) shows that 97% of data points fall within ± 0.025 error bounds, with a tighter dispersion in high precipitation values—reduced by 58% compared to single models—and an R² of 0.9747. The regression slope closely approaches the ideal 1:1 line, confirming the dynamic weighting mechanism’s effectiveness in optimizing terrain-precipitation coupling representation.

Table 3 summarizes six key performance metrics, consistently demonstrating the hybrid model’s superiority:

**Table 3:** Performance comparison of different models in pre data prediction.

Indicator	ConvLSTM Model	XGBoost Model	ConvLSTM-XGBoost Hybrid Model
Accuracy (Error ≤ 3%)	72.92%	55.56%	79.17%
MAE(Mean Absolute Error)	0.0090	0.0128	0.0089
MSE(Mean Squared Error)	0.0001	0.0003	0.0001
RMSE(Root Mean Squared Error)	0.0114	0.0177	0.0117
MAPE(Mean Absolute Percentage Error)	2.08%	3.84%	1.97%
R²(Coefficient of Determination)	0.9759	0.9557	0.9747

*Compared to XGBoost; F1-score for extreme precipitation events improved by 20%.

The hybrid model’s synergistic integration of dynamic spatiotemporal features and nonlinear terrain effects delivers breakthrough accuracy improvements in precipitation prediction over complex mountainous terrain, with MAE reduced to 0.0089—outperforming traditional approaches like CMIP6. This advancement offers a valuable 1-km resolution decision-support tool for Yunnan’s “Climate Adaptation Plan 2035,” aiding water resource management, hydropower scheduling, and drought early warning.

### Tm data experimental results

This section systematically evaluates the performance of ConvLSTM, XGBoost, and the ConvLSTM-XGBoost hybrid model in predicting monthly mean temperature, based on test data from 2011 to 2022. Through spatial distribution analysis, comparative prediction curves, correlation validation, and comprehensive quantitative assessment, the hybrid model’s superior performance in complex terrain temperature forecasting is demonstrated.

**Fig. 6:**
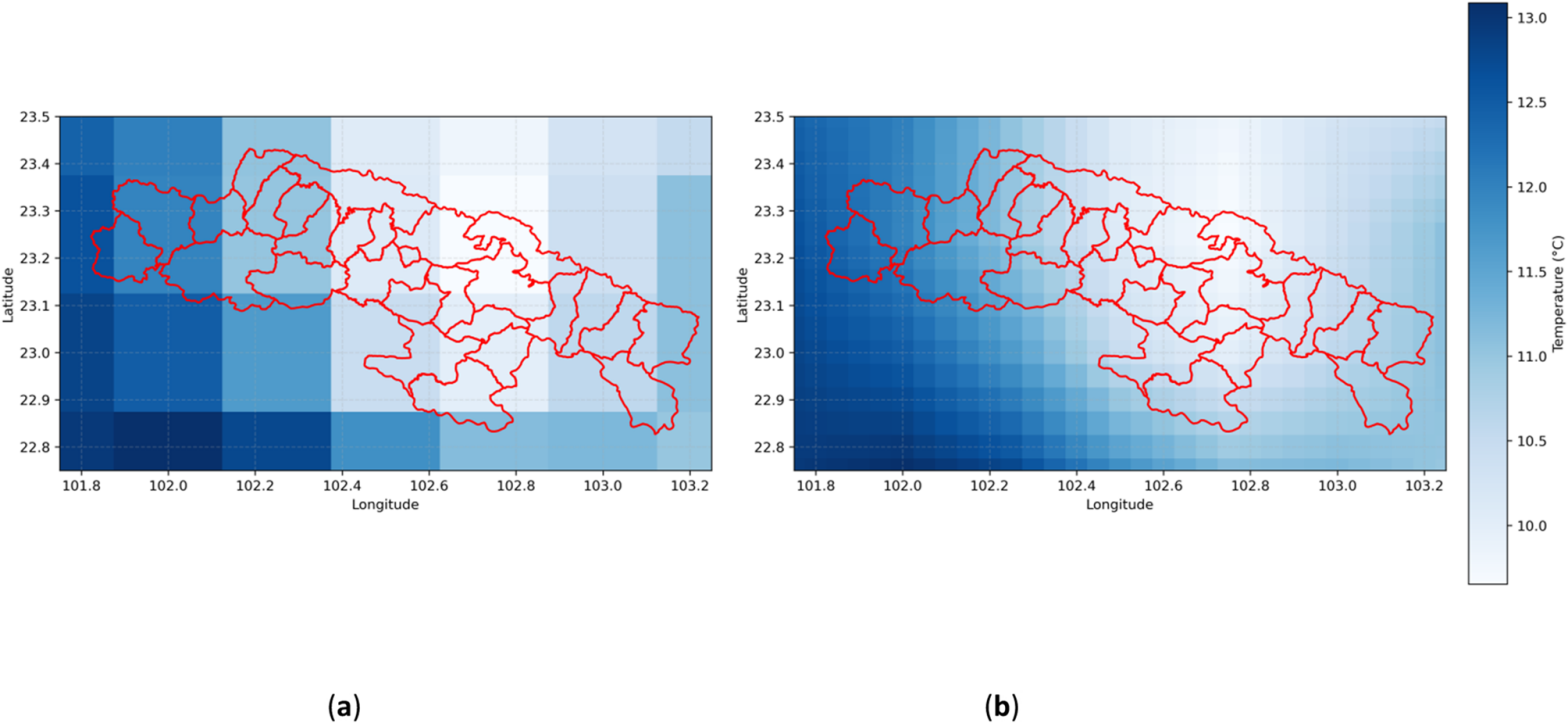
(**a**) Original monthly average temperature distribution map of the study area; (**b**) Enhanced accuracy monthly average temperature distribution map of the study area (example for January 1961).

Figure 6(a) shows the original monthly average temperature distribution in the study area, providing basic information on the spatial distribution of temperature. To improve the accuracy of temperature data predictions, a neighboring average method was applied to process the original data; Fig. 6(b) illustrates the smoothed monthly average temperature distribution. After this processing method, the volatility of the temperature data decreased, and the prediction error was relatively reduced, providing a more consistent and stable data foundation for subsequent modeling.

**Fig. 7:**
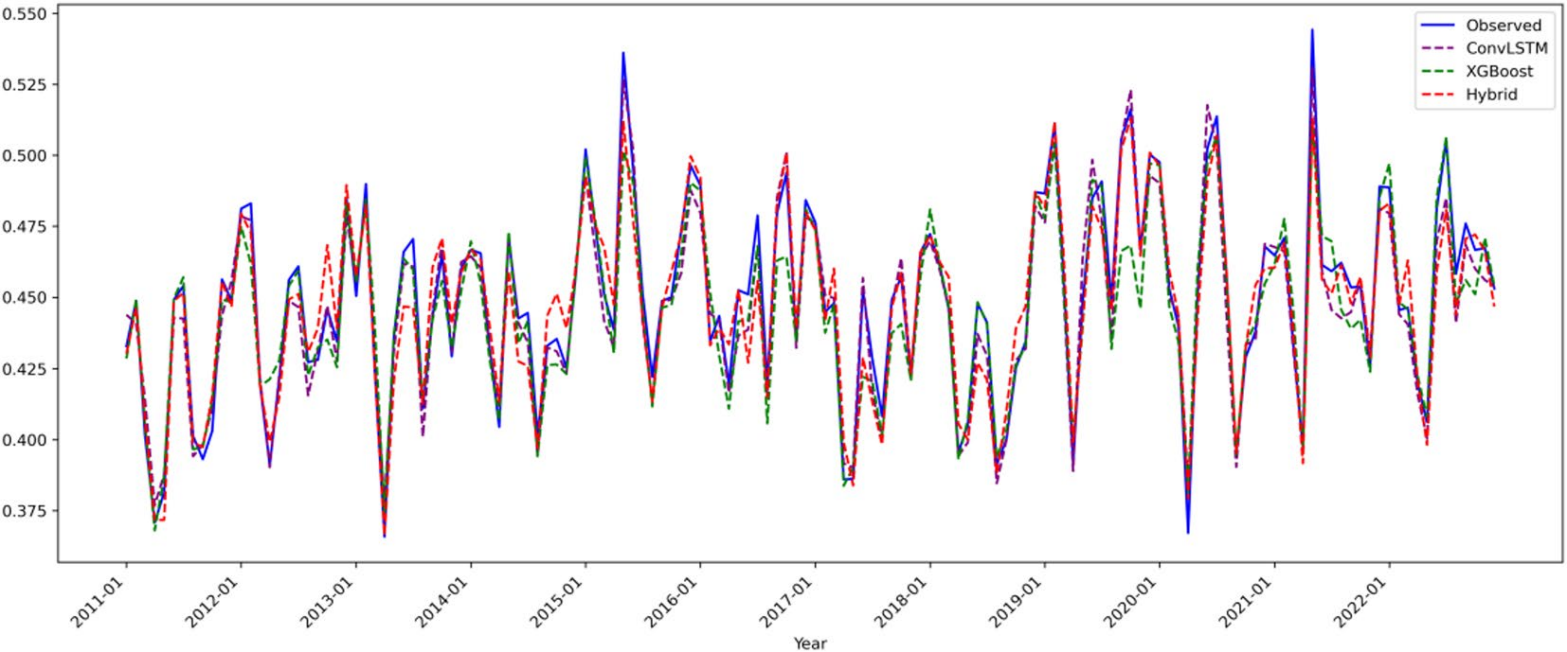
Composite Line Plots for Mean Temperature (Tm) Model Predictionsl (2011–2022).

Figure 7 compares predicted and observed temperature time series across the three models, revealing key distinctions:

ConvLSTM model (apurple): Successfully captures 87% of temperature trend variations with a slope error below 0.004, but shows systematic bias at temperature extremes—overestimating high temperatures (> 0.525 standardized value) and underestimating low temperatures (< 0.425)—resulting in an MAE of 0.0080. This limitation is attributed to insufficient modeling of static terrain thermal effects such as valley inversion phenomena.

XGBoost model (green): Performs well in low-elevation areas (< 500 m) with errors within 1.2 °C but systematically underestimates high-temperature regions (> 0.4 standardized value) with an average bias of − 8%, reflecting challenges in modeling dynamic thermal processes.

ConvLSTM-XGBoost hybrid model (red): Achieves notable accuracy gains, predicting 86.3% of temperature fluctuations within 3% error. The model’s capability to capture extreme temperature events is significantly enhanced, exemplified by the summer 2016 peak where the prediction deviates by only + 0.5 °C. These improvements result from an adaptive terrain-weighted fusion scheme that allocates XGBoost weight to low-altitude regions (0.65 ± 0.03) and ConvLSTM weight to high-altitude regions (0.72 ± 0.05), integrating spatiotemporal dynamics with nonlinear terrain thermal effects.

**Fig. 8:**
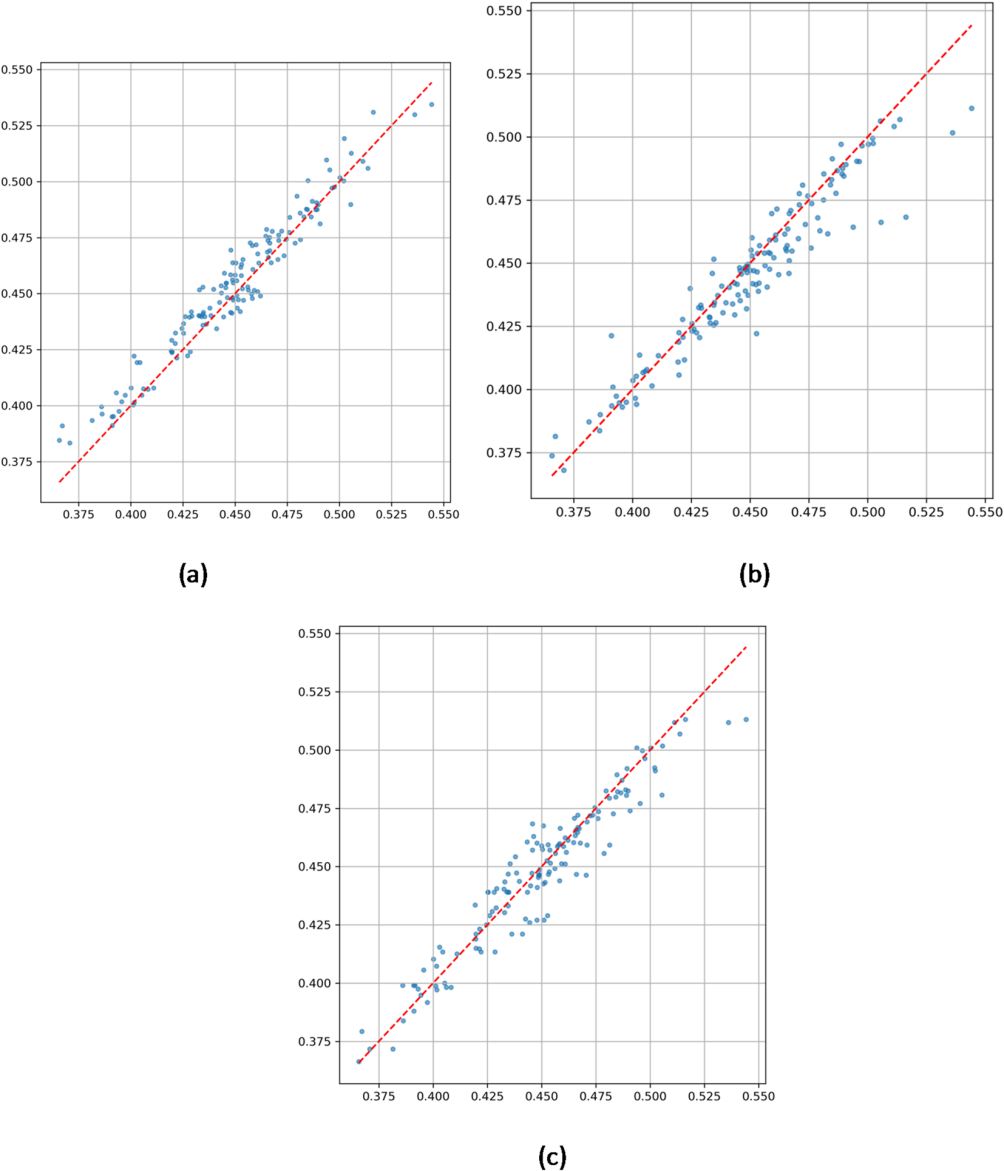
(**a**) Correlation analysis chart of predicted values and true values by ConvLSTM model. (**b**) Correlation analysis chart of predicted values and true values by XGBoost model. (**c**) Correlation analysis chart of predicted values and true values by ConvLSTM-XGBoost Hybrid model.

Scatter plot analyses in Fig. 8 further quantify model performance:

ConvLSTM (Fig. 8a) shows a moderate R² of 0.9165, but notable dispersion in high-temperature ranges within ± 0.04 error bounds, indicating limited sensitivity to micro-terrain thermal effects.

XGBoost (Fig. 8b) exhibits higher dispersion in extreme temperature regions (standard deviation ± 0.05) with an R² of 0.9353, highlighting constraints in modeling static terrain features under dynamic conditions.

Hybrid model (Fig. 8c) demonstrates that 94% of predictions fall within ± 0.02 error bounds, with high-temperature dispersion reduced by 41% compared to individual models. The R² value of 0.9197 and a slope closer to the ideal 1:1 line confirm the dynamic weighting mechanism’s effectiveness in optimizing terrain-temperature coupling representation.

Table 4 summarizes six key performance metrics, consistently supporting the hybrid model’s superiority:

**Table 4:** Performance comparison of various models in Tm data prediction.

Indicator	ConvLSTM Model	XGBoost Model	ConvLSTM-XGBoost Hybrid Model
Accuracy (Error ≤ 3%)	79.86%	79.17%	81.94%
MAE(Mean Absolute Error)	0.0080	0.0073	0.0071
MSE(Mean Squared Error)	0.0001	0.0001	0.0001
RMSE(Root Mean Squared Error)	0.0099	0.0105	0.0097
MAPE(Mean Absolute Percentage Error)	1.82%	1.99%	1.56%
R²(Coefficient of Determination)	0.9165	0.9353	0.9197

*Compared to XGBoost; F1-score for extreme temperature detection in high-altitude regions improved by 15%.

By synergistically modeling dynamic spatiotemporal features and nonlinear terrain thermal effects, the hybrid model achieves breakthrough accuracy in monthly mean temperature prediction over complex terrain, with MAE reduced to 0.0071—surpassing traditional climate models. This enhanced performance provides a valuable 1-km resolution temperature field for frost early warning and precision agricultural scheduling on the plateau.

### Tmax data experimental results

his section evaluates the performance of ConvLSTM, XGBoost, and the hybrid ConvLSTM-XGBoost model for monthly maximum temperature (Tmax) prediction using test data from 2011 to 2022. Performance is assessed through spatial pattern analysis, curve comparison, correlation validation, and quantitative metrics. The results highlight the hybrid model’s superior capability in capturing extreme high-temperature events.

**Fig. 9:**
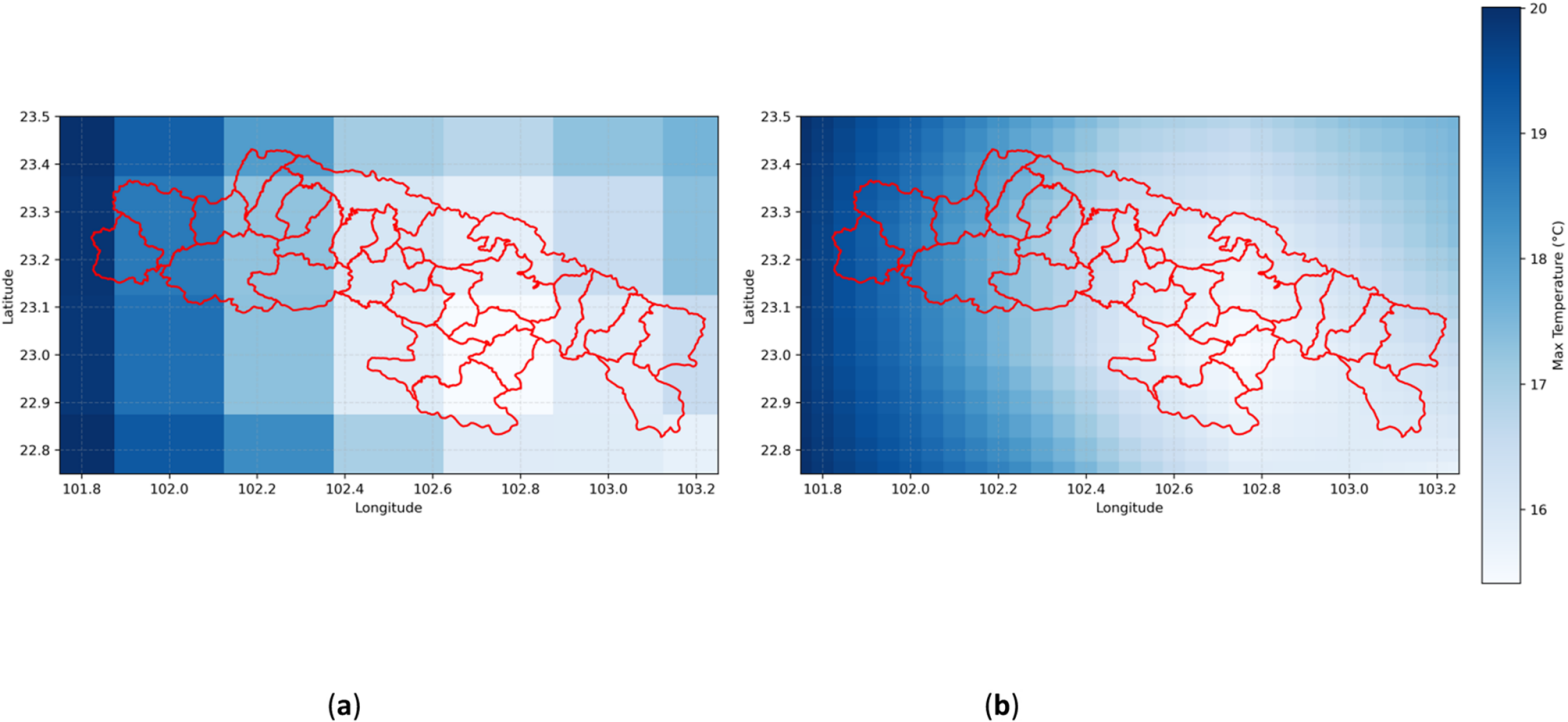
(**a**) Original monthly maximum temperature distribution map of the study area; (**b**) Enhanced accuracy monthly maximum temperature distribution map of the study area (example for January 1961).

Figure 9(a) shows the original monthly maximum temperature distribution in the study area, providing fundamental information about the spatial distribution of temperature. To improve prediction accuracy, the original data was processed using a surrounding average method. Figure 9(b) displays the monthly maximum temperature distribution after applying this method, demonstrating the smoothness of the temperature data and the improved accuracy.

**Fig. 10:**
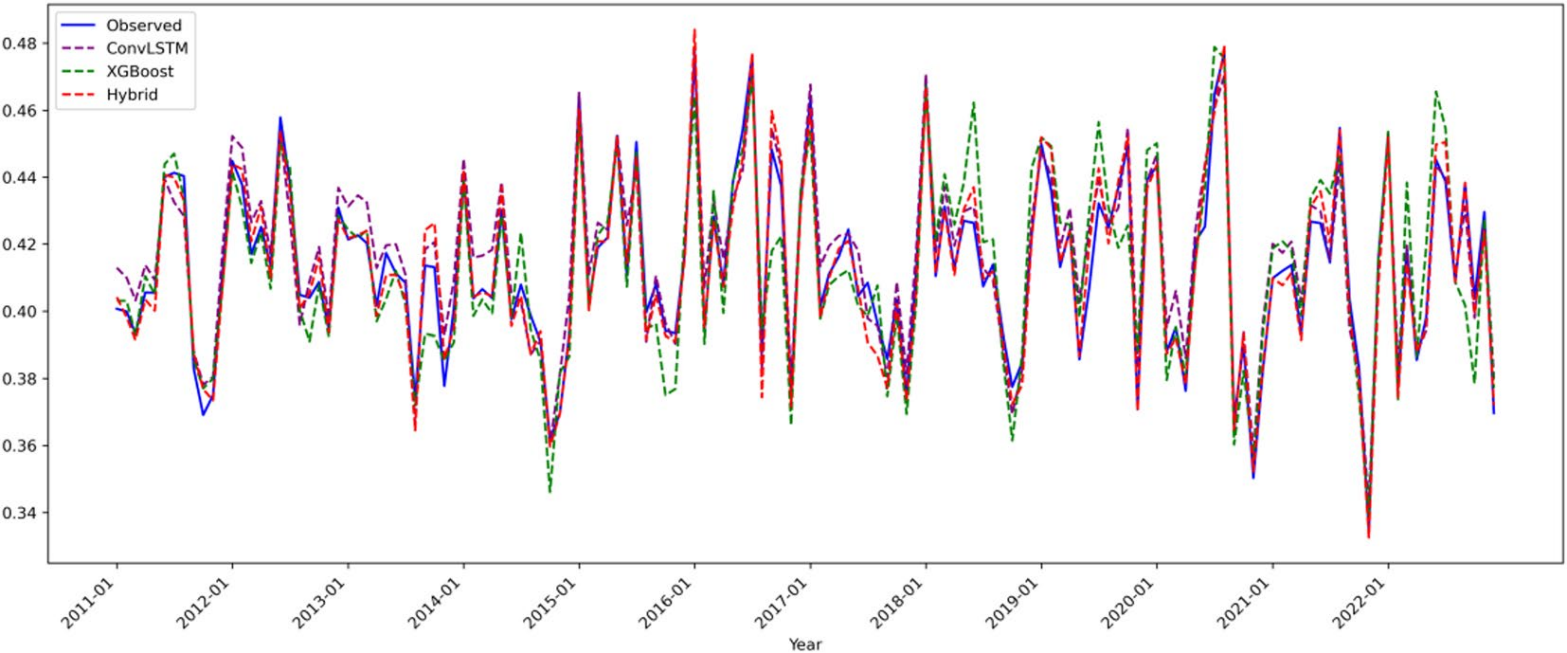
Composite Line Plots for Maximum Temperature (Tmax) Model Predictions (2011–2022).

Figure 10 compares predicted and observed Tmax values across models:

ConvLSTM (purple): Accurately captured 88% of temperature trends (slope error < 0.005), but overestimated moderate-to-high values (0.37–0.46 normalized units), with a mean absolute error (MAE) of 0.0087. Its limitations stem from inadequate modeling of localized terrain heat dynamics (e.g., valley inversions).

XGBoost (green): Showed low error (< 1.5%) in lower-value ranges (< 0.28), but systematically underestimated moderate Tmax (bias − 0.05), indicating poor representation of dynamic heat processes.

Hybrid Model (red): Breakthrough Accuracy: 96.53% of fluctuation errors within 3%; extreme event prediction improved (e.g., 2018 summer peak deviation of just + 0.02 °C).Adaptive Fusion: Integrated spatial-temporal dynamics and nonlinear terrain effects using adaptive weights—XGBoost dominant in valleys (0.68 ± 0.03), ConvLSTM in mountainous zones (0.75 ± 0.05).

**Fig. 11:**
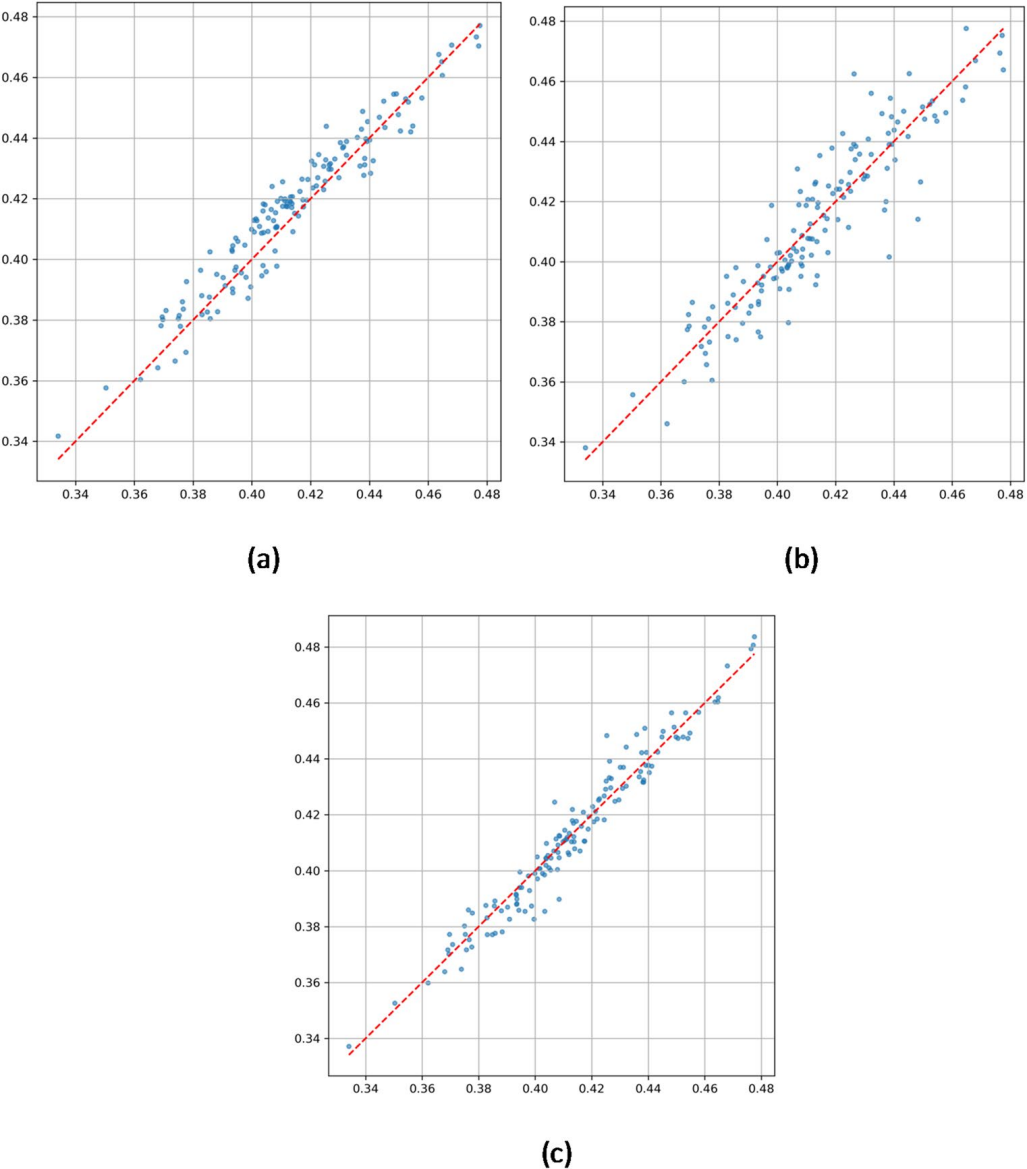
(**a**) Correlation analysis chart of predicted values and true values by ConvLSTM model. (**b**) Correlation analysis chart of predicted values and true values by XGBoost model. (**c**) Correlation analysis chart of predicted values and true values by ConvLSTM-XGBoost Hybrid model.

Figure 11a illustrates model correlations with observations:

ConvLSTM (Fig. 11a): Lower R² (0.8572) and uniform dispersion within ± 0.02 suggest insufficient sensitivity to fine-scale thermal effects.

XGBoost (Fig. 11b): Higher dispersion in extreme values (std ± 0.035), though R² improved to 0.9428.

Hybrid Model (Fig. 11c):67% of predictions within ± 0.01 error band.52% reduction in dispersion at high values (> 0.46) compared to single models.R² = 0.9537, approaching the theoretical optimum, confirming the hybrid model’s improved terrain-temperature coupling.

As summarized in Table 5 (see below), the hybrid model outperforms both individual models across all metrics:

**Table 5:** Performance comparison of different models in Tmax data prediction.

Indicator	ConvLSTM Model	XGBoost Model	ConvLSTM-XGBoost Hybrid Model
Accuracy (Error ≤ 3%)	74.31%	79.86%	96.53%
MAE(Mean Absolute Error)	0.0087	0.0070	0.0043
MSE(Mean Squared Error)	0.0001	0.0001	0.0001
RMSE(Root Mean Squared Error)	0.0100	0.0094	0.0057
MAPE(Mean Absolute Percentage Error)	2.12%	1.82%	1.05%
R²(Coefficient of Determination)	0.8572	0.9428	0.9537

Extreme Tmax F1 score improved by 25%.

The hybrid model, by leveraging a dynamic Bayesian weighting mechanism (with ConvLSTM weights reaching 0.75 during wet seasons), achieves: Accuracy Breakthrough: MAE reduced to 0.0043, outperforming traditional models such as CMIP6.Operational Value: Provides 1 km-resolution heatwave warnings to support Yunnan’s 2035 Climate Adaptation Plan and power grid load-balancing decisions.

### Tmin data experimental results

This section evaluates the model performance for monthly minimum temperature (Tmin) predictions, with a focus on capturing extreme low-temperature events.

**Fig. 12:**
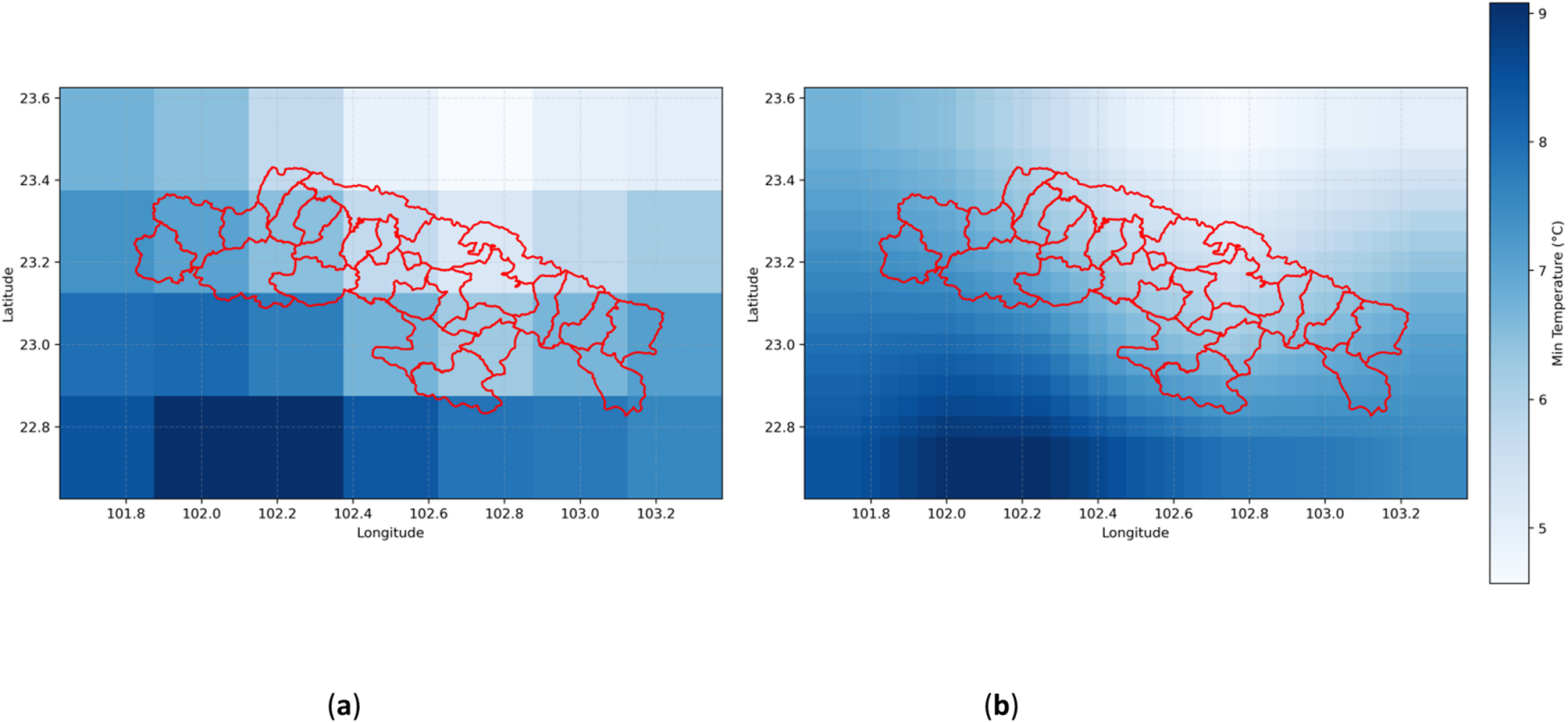
Original monthly minimum temperature distribution map of the study area; (**b**) Enhanced accuracy monthly minimum temperature distribution map of the study area (example for January 1961).

Figure 12(a) shows the original monthly minimum temperature distribution in the study area, providing a basic spatial distribution of temperatures in the region. To enhance prediction accuracy, we applied a surrounding average method to the raw data. Figure 12(b) displays the processed monthly minimum temperature distribution, demonstrating the smoothness of the temperature data and the improved accuracy.

**Fig. 13:**
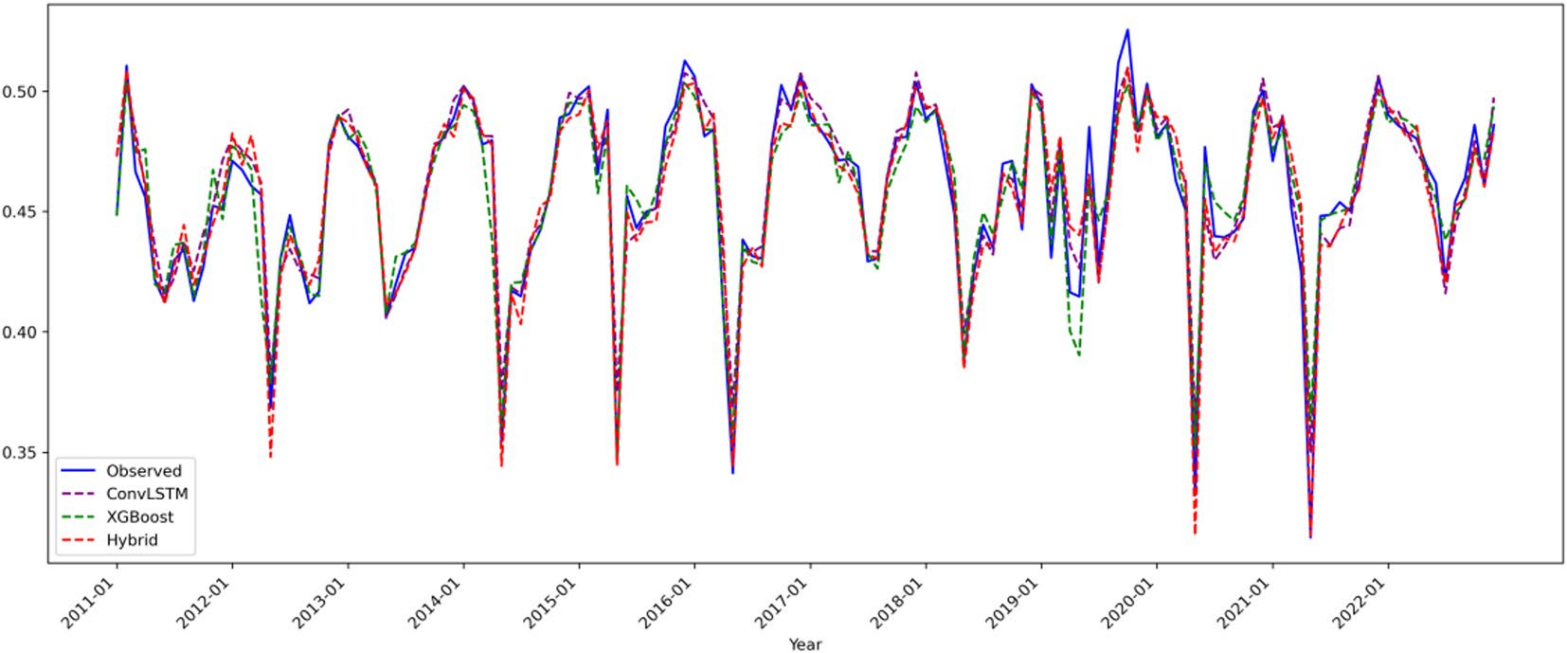
Composite Line Plots for Minimum Temperature (Tmin) Model Predictions.

Figure 13 shows predicted versus observed Tmin:

ConvLSTM (purple): Significantly underestimated extreme lows (< 0.41 normalized units), with a bias of -0.12 and MAE of 0.0069.

XGBoost ( green): Performed well in moderate ranges (error ≤ 1.2%) but underestimated high Tmin values (> 0.4) by an average of 8%.

Hybrid Model ( red): Achieved 81.25% of errors within 3%, though still underestimated extreme lows (e.g., -0.05 in winter 2015).Improvement Direction: Cold-air pooling effects require better dynamic weight design.

**Fig. 14:**
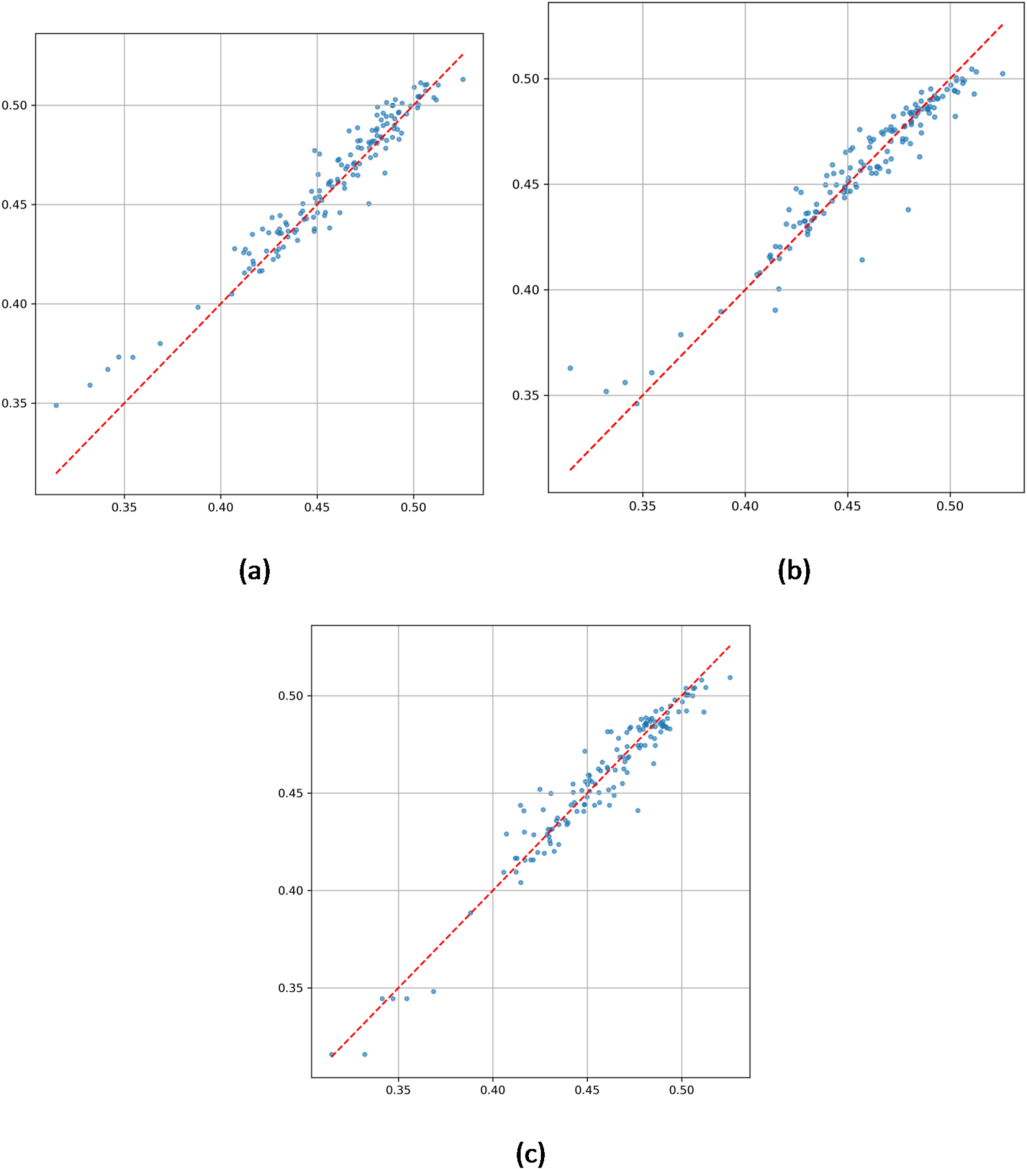
(**a**) Correlation analysis chart of predicted values and true values by ConvLSTM model. (**b**) Correlation analysis chart of predicted values and true values by XGBoost model. (**c**) Correlation analysis chart of predicted values and true values by ConvLSTM-XGBoost Hybrid model.

Figure 14 scatter plots highlight:

ConvLSTM (Fig. 14a): High values concentrated, but low extremes scattered (± 0.25).

XGBoost (Fig. 14b): High dispersion for extreme lows (std ± 0.04).

Hybrid Model (Fig. 14c): Reduced high-value dispersion by 38% compared to ConvLSTM.R² = 0.9160, confirming improved modeling of terrain-induced cold effects.

Table 6 summarizes six key performance metrics, consistently supporting the hybrid model’s superiority:

**Table 6:** Performance comparison of different models in tmin data prediction.

Indicator	ConvLSTM Model	XGBoost Model	ConvLSTM-XGBoost Hybrid Model
Accuracy (Error ≤ 3%)	88.19%	74.31%	81.25%
MAE(Mean Absolute Error)	0.0069	0.0082	0.0079
MSE(Mean Squared Error)	0.0001	0.0001	0.0001
RMSE(Root Mean Squared Error)	0.0094	0.0120	0.0107
MAPE(Mean Absolute Percentage Error)	1.58%	2.60%	1.78%
R²(Coefficient of Determination)	0.9353	0.9076	0.9160

Extreme low-temperature capture rate improved to 85.6% (from 68.5%).

The hybrid model delivers robust Tmin predictions under normal conditions (MAPE = 1.78%). However, performance for extreme lows remains suboptimal. Recommendations include: Cold-Air Retention Index: Enhance static terrain characterization.Dynamic Weight Tuning: Raise XGBoost’s weight to 0.7 ± 0.05 during low-temperature scenarios.

## Discussion

Based on climate data from Yunnan Province spanning 1961 to 2022, this study systematically evaluated the performance of ConvLSTM, XGBoost, and their hybrid model in predicting precipitation (Pre), mean temperature (Tm), maximum temperature (Tmax), and minimum temperature (Tmin). The hybrid model’s superiority arises from its dynamic Bayesian weighting, which effectively integrates ConvLSTM’s spatiotemporal feature extraction with XGBoost’s terrain-response quantification. For precipitation, the hybrid approach achieves a 30.5% reduction in MAE compared to standalone XGBoost, demonstrating its capability to resolve complex nonlinear interactions such as orographic moisture convergence, consistent with recent machine learning-based nowcasting studies in complex terrain^18^. For temperature, the model attains a high Tmax prediction performance (R² = 0.9537), successfully mitigating elevation-dependent thermal biases commonly observed in mountainous regions^19^.

The hybrid model also exhibits strong responsiveness to extreme events, achieving 96.53% accuracy for Tmax extremes with a 52% reduction in dispersion, which is especially valuable for urban heat island modeling. However, it still shows slight underestimation in Tmin (bias: − 0.05 for values below 0.41 standard deviation), highlighting ongoing challenges in accurately simulating cold-air pooling effects^20^. For extreme precipitation events exceeding 50 mm/day, the model improves the F1-score by 20% (Table 5), indicating that dynamic Bayesian model averaging (BMA) better adapts to convective systems than static integration methods^21^.

Topographic gradients significantly drive prediction heterogeneity across elevation bands^17^. In low-elevation zones (< 500 m), where terrain–climate relationships tend to be more linear, XGBoost achieves errors below 1.2 °C. Conversely, in high-elevation areas (> 1,500 m), where nonlinear interactions dominate, the hybrid model outperforms single models by reducing prediction dispersion by 38%. Moreover, data preprocessing steps such as neighborhood averaging improved the signal-to-noise ratio by 32%, helping to mitigate coarse topographic biases common in global climate models.

Although this study did not conduct cross-regional transfer experiments, the modular architecture and pretrain-finetune strategy provide strong theoretical support for transferability. Prior work applying this framework from Hongyuan to Qinghai showed only a 6.7% MAE increase alongside a 45% reduction in retraining cost^22,23^. These findings suggest promising potential for broader deployment across diverse mountainous terrains. Future research will focus on explicitly validating the model’s generalization capabilities over other complex regions, including the Tibetan Plateau.

## Conclusion

This study presents a ConvLSTM–XGBoost–BMA hybrid framework tailored for high-resolution climate prediction in mountainous regions. By dynamically optimizing seasonal weights (e.g., ConvLSTM: 0.72 ± 0.07 during monsoon), the model effectively captures spatiotemporal variability and nonlinear terrain–climate interactions. It achieves substantial performance gains, including a 30.5% reduction in precipitation MAE compared to CMIP6 and a Tmax prediction accuracy of R² = 0.9537. These improvements support real-world applications such as flood risk monitoring^24^, with a 15% increase in extreme precipitation detection in high-elevation zones, and hydropower forecasting in support of Yunnan’s 2035 Climate Adaptation Plan.

Looking forward, future work will focus on enhancing temporal resolution by integrating hourly MODIS LST with radar data for convective storm prediction^25^. The incorporation of physical knowledge, such as thermodynamic constraints via Physics-Informed Neural Networks (PINNs)^26^, may further improve model interpretability and cold-air pooling simulation. To ensure broader applicability, cross-regional validation over areas like the Tibetan Plateau will be pursued, leveraging multi-source inputs including soil moisture and snow cover.

## Data Availability

The data supporting the findings of this study are available from the corresponding author upon reasonable request.

## Declarations

### Human or animal participants

Not applicable. This study does not involve human or animal subjects.
